# Inter-organizational knowledge and information transfer in long-term care for older persons: Do sector and resource availability matter?

**DOI:** 10.1108/JHOM-11-2024-0453

**Published:** 2025-04-10

**Authors:** Rūta Kazlauskaitė, Irina Liubertė, Virginija Poškutė, Irmina Matonytė

**Affiliations:** ISM University of Management and Economics, Vilnius, Lithuania; General Jonas Žemaitis Military Academy of Lithuania, Vilnius, Lithuania

**Keywords:** Knowledge and information transfer, Social capital, Resource availability, Long-term care

## Abstract

**Purpose:**

This article aims to determine if and how social capital enables inter-organizational knowledge and information transfer in long-term care (LTC) for older persons and within what boundary conditions.

**Design/methodology/approach:**

The study builds on a survey of key LTC actors (*N* = 265) representing public, private and non-governmental organizations.

**Findings:**

Our findings revealed a positive relationship between two social capital dimensions and knowledge and information transfer. In addition, partial support was provided for the moderating effects of sector and resource availability.

**Originality/value:**

Our study explores to what extent social capital facilitates inter-organizational knowledge and information transfer in LTC. Secondly, it contributes to the broader knowledge management literature by disclosing two boundary conditions in the above relationship.

## Introduction

With EU populations aging rapidly, the number of older individuals with long-term care (LTC) needs is projected to reach 38.1 million by 2050 (European Commission, 2021a). The supply and quality of formal care services for older people are already insufficient. Inter-organizational collaboration is increasingly considered critical in maintaining and improving accessibility and quality of formal LTC (Simons *et al*., 2022; European Commission, 2021a; Lørum and Smith, 2024), as responsibilities for LTC services are split between health and social care domains or funded from different (national or local) levels in many countries across Europe (Spasova *et al*., 2018; European Commission, 2021b), calling for research on the facilitators of inter-organizational collaboration (Yoshida *et al*., 2024; van der Schors *et al*., 2021).

Knowledge and information transfer is a major facilitator and a form of inter-organizational collaboration in knowledge-intensive LTC (Doornebosch *et al*., 2022; Hujala and Laihonen, 2021), where single organizations may not provide entire care on their own, nor are they competent or knowledgeable of all aspects of health and social care, and where insufficient knowledge and information may compromise health and care (Gonçalves *et al*., 2022). Prior LTC research has focused on knowledge and information sharing within organizational boundaries (Kosklin *et al*., 2023) or in integrated care teams (Rydenfält *et al*., 2024; Doornebosch *et al*., 2022). In this article we address knowledge and information transfer between LTC-related organizations and examine their social capital as a potential facilitator.

Traditionally formal LTC was mostly provided by public organizations in the European Union (EU), except for Germany and the Netherlands, where the private sector is the major LTC provider (Riedel *et al*., 2016). Currently the public sector may no longer meet the growing demand, thus LTC services are also provided by private (non-profit and for-profit) and non-governmental organizations, though with variations among EU countries (European Commission, 2021a). Countries also vary in regards to their history of private and non-governmental organization involvement in LTC, which is a relatively recent phenomenon in the new EU member states (Schulmann *et al*., 2014). Against this background, inter-organizational knowledge and information transfer in LTC is becoming even more critical and complex than in other industries. Research on sector-specific enablers of knowledge and information transfer in LTC remains scant (Zbuchea *et al*., 2020; Carlini *et al*., 2023).

To address the above gaps, this article aims to determine if and how social capital enables knowledge and information transfer in LTC organizations and within what boundary conditions. In doing so, we build on the theories of social capital and interpersonal behavior. Although people of all ages may require LTC, in this study we focus on LTC for older people, which refers to a range of personal, social and medical services related to the help in daily living activities and/or permanent nursing care, provided at one’s home or in institutionalized settings. Based on the results of a survey of key LTC actors from public, private and non-governmental organizations in Lithuania, our findings show that the structural and relational dimensions of social capital facilitate inter-organizational knowledge and information transfer in LTC. Secondly, this relationship is partially moderated by the sector and resource availability.

## Theoretical background and hypotheses

### Inter-organizational knowledge and information transfer in LTC

In a broad sense inter-organizational knowledge and information transfer (also referred to as knowledge and/or information sharing in literature) may be defined as a process through which organizations share, receive and are affected by knowledge and information received from others (Van Wijk *et al*., 2008). In health and social care, it is believed to lead to improved service accessibility and quality (Radević *et al*., 2023; Pedersen *et al*., 2023); however, it is more complex than in other industries, as it involves organizations assuming different roles. While healthcare organizations deal with health issues and the work of health professionals, those in the social sector provide support to older people in their daily activities and organize the work of social workers. The first ones are usually funded at the national, and the latter at the national, regional or local levels (European Commission, 2021a). LTC actors also represent different occupations and sectors, which vary in perceived public status, aims, levels of power, competence and resources, etc., which may inhibit their understanding of the roles and abilities of other organizations and lead to transfer failure (Auschra, 2018; Lyng *et al*., 2024).

### Social capital and inter-organizational knowledge and information transfer

Social capital theory has been used in recent knowledge management research in different domains (e.g. Zhao and Detlor, 2023; Santos *et al*., 2023). Its basic premise is that networks of more or less institutionalized relationships constitute valuable resources facilitating collective actions (Adler and Kwon, 2002; Bourdieu, 1985). It distinguishes between individual and collective (e.g. organizational) capital (Coleman, 1988; Putnam, 1993). This article follows the collective perspective, widely used in health and social care literature (Derose and Varda, 2009). Social capital is a three-dimensional concept referring to existing and prospective resources embedded within a network of relationships and available through them (Nahapiet and Ghoshal, 1998). Its *structural* dimension relates to relationship configuration and strength, which provide opportunity to access resources (Nahapiet and Ghoshal, 1998). The *relational* dimension refers to the nature of relationships deriving from interaction between network ties, such as trust and reciprocity (Coleman, 1988). Finally, the *cognitive* dimension relates to shared systems of meaning among network ties (Nahapiet and Ghoshal, 1998).

Prior research has built on social capital theory a lot to explain social capital effects on knowledge transfer; yet often such studies focused on selected social capital dimensions (Lee *et al*., 2023; McFadyen and Cannella, 2004). We follow the above multi-dimensional perspective and propose that social capital facilitates knowledge and information transfer in multiple ways (Adler and Kwon, 2002; Maurer *et al*., 2011; Santos *et al*., 2023). First, social relations of organizational members provide opportunity for knowledge transfer and more frequent interactions with other network members, which strengthen existing relations, that at the start may be weak (Oh and Bush, 2016). Thus, larger networks and more frequent and closer interaction provide a wider scope for knowledge transfer between organizations (Ferrer-Serrano *et al*., 2022; Hemmert, 2019).

Secondly, knowledge and information transfer require trust in other network members (Levin and Walter, 2019; Ferrer-Serrano *et al*., 2022). Trust is critical in LTC, where actors vary in perceived public status, professional values, competences, problem solving approaches, etc. (Song *et al*., 2019; Gonçalves *et al*., 2024; Yadav *et al*., 2025). Thus, trust in other network members’ ability to perform their roles and willingness to reciprocate are indispensable. Trust instils belief in careful and appropriate handling of the acquired information and knowledge by others and leads to enhanced reciprocity, which also facilitates inter-organizational knowledge and information transfer (Ganguly *et al*., 2019).

Finally, inter-organizational knowledge and information transfer necessitate a shared vision, as a lack of mutual understanding between organizations may inhibit their willingness and ability to work together (Lee *et al*., 2023). The pursuit and implementation of a shared vision require the development of a common culture, or shared values and interests (Selsky and Parker, 2005; Levin and Walter, 2019). These are critical in LTC where engagement of private, public and non-governmental organizations may be driven by different motivations, and characterized by different cultural and professional values and specializations (Auschra, 2018).

Prior research in other industries has yielded evidence in support of the relationship between some dimensions of social capital and knowledge and information transfer too (e.g. Miković *et al*., 2020; Santos *et al*., 2023; Rodríguez-Aceves *et al*., 2023; Lee *et al*., 2023).

Hence, we propose the following hypothesis:

H1. (a) Structural (number of relations with other LTC actors and frequency of interaction with them), (b) relational (trust and reciprocity), and (c) cognitive dimensions (shared vision and values) of social capital are positively related to knowledge and information transfer.

### Moderating effects of the sector

In Europe LTC services are provided by public (governmental/municipal), private (for-profit and non-profit) and non-governmental (NGO) organizations [1]. Knowledge and information sharing among different sector organizations is complex as it may be driven by different factors stemming from varying macro-economic and organizational contexts (Susha *et al*., 2023; Bloice and Burnett, 2016). In this paper we explore the role of such organizational elements as structure and culture and their relationship with social capital.

If compared to private and non-governmental organizations, public organizations are typically more bureaucratic, with higher hierarchical structures and larger degrees of centralization and formalization (Reynaers and van der Wal, 2018), which impedes knowledge and information transfer (Radević *et al*., 2023). Knowledge transfer faces more constraints in the public sector. Its employees often associate knowledge with power, which reduces their willingness to share it for fear of losing it (Yang and Maxwell, 2011). Loss of power has been acknowledged as a critical barrier to knowledge transfer (Zhao and Detlor, 2023). In addition, public organizations often have vague, competing and difficult to measure goals and diverse values, which further complicates working on a common task with others (Hujala and Laihonen, 2023). In contrast to private organizations, where knowledge management is centered on the organization and its performance, public organizations need to take the broader societal context into consideration (Laihonen *et al*., 2024). As regards NGOs, their activities are primarily driven by social values and genuine concern about service users (Dickinson *et al*., 2012), and differences in others’ interests (e.g. profit-making in private organizations) may diminish NGO trust in them. NGOs may also be reluctant to partner with public organizations due to fear of increased bureaucracy and reduced flexibility and autonomy (Abendstern *et al*., 2018). NGOs also tend to have stronger relationships with local communities and society than other stakeholders, local government in particular (Gazley, 2010). Local governments play a critical role in LTC; thus, having none or limited prior experience of working with them precludes both trust and willingness to engage in knowledge transfer. To conclude, we expect these differences in organizational structure and culture to weaken the relationship between social capital and knowledge transfer in public and non-governmental organizations in comparison to private ones. Respectively, we propose the following hypothesis:

H2. Sector moderates the relationship between (a) structural, (b) relational, and (c) cognitive dimensions of social capital and knowledge and information transfer, so that the relationship is the strongest in private and the weakest in NGOs.

### Moderating effects of resource availability

The theory of interpersonal behavior (Triandis, 1977), proposes that human behavior is a function or intention, habit and facilitating conditions, i.e. factors whose presence or absence determine the difficulty or ease of performing a particular behavior and engaging in it. Availability of resources (e.g. time, money, etc.) needed to engage in a particular behavior is viewed as one of such facilitating conditions (Koay *et al*., 2022; Taylor and Todd, 1995), while lack of resources is a significant constraint. Respectively, we argue that the relationship between social capital and knowledge transfer will be stronger when resources are available and *vice versa*. This theory has been applied in prior knowledge management research (Koay *et al*., 2022; Jeon *et al*., 2011).

Lack of resources has been identified as an important barrier to cross-sectoral knowledge and information transfer (Susha *et al*., 2023; Alderwick *et al*., 2021). LTC actors mainly represent public organizations, NGOs, and smaller private firms, which traditionally have more limited resources (Woschke *et al*., 2017). Furthermore, resource availability is not uniform across sectors. Public health and social care organizations, mostly local municipal organizations, traditionally have more limited financial resources in comparison to private ones (Torchia *et al*., 2015; Sunnemark *et al*., 2024). NGOs, meanwhile, are subject to high volatility in funding opportunities (Zbuchea *et al*., 2020), heavily rely on donors (Miković *et al*., 2020) and volunteers (Curado *et al*., 2023), and have lesser competent human resources (Poškutė *et al*., 2022). Hence NGOs are more likely to have more limited resources needed for knowledge and information transfer in comparison to their public and private counterparts. On the other hand, NGOs may draw significant donations and volunteers through their extended community networks, which explains their ability to work effectively even with limited resources (Schneider, 2009). Prior research also showed that the NGO size was not related with knowledge transfer and collaboration (Kim and Peng, 2018); instead, employee altruism was found as an important predictor of knowledge sharing in NGOs (Curado *et al*., 2023), making a potential substitute for scarce resources. In addition, what makes NGOs distinct from other sectors is that their activities are largely driven by other than financial objectives (Enjolras and Sivesind, 2018). NGOs are likely to attach higher importance to their mission of taking care and be more willing to “go out of their way to help service users” (Dickinson *et al*., 2012, p. 14). In addition NGOs may be required by their donors to follow transparent procedures and humanitarian and ethical policies, which may not be the case in private organizations (Moshtari and Vanpoucke, 2021). Where NGOs seek to create social value for their wider communities and society, this vision motivates their external knowledge sharing (Bloice and Burnett, 2016). Hence, we propose the following hypothesis:

H3. The relationship between (a) structural, (b) relational, and (c) cognitive dimensions of social capital and knowledge and information transfer is moderated by sector and resource availability, so that the relationship between social capital and knowledge and information transfer is stronger in public and private sector organizations with a higher degree of resource availability. The effect of resource availability in NGOs will be weaker.

## Methods

### Research context

This study was conducted in Lithuania with shrinking [2] and fast aging [3] population and large numbers of older people at risk of poverty or social exclusion [4]. LTC responsibilities here are shared horizontally by health and social care sectors (Eurocarers, 2021), commonly to many other countries in the EU, and require a better integration, as healthy life expectancy among older persons is considerably lower than the EU average. Integrated care services are relatively new and underdeveloped. Informal care has traditionally been most favored, like in other Eastern and Southern European countries; however, the demand for institutional services has grown due to a dramatic rise in old-age dependency ratio and retirement age (Poškutė and Greve, 2017). Formal LTC is insufficient and its quality is inadequate (Spasova *et al*., 2018). LTC services are provided by all three sectors; however, municipal providers prevail. Inter-organizational collaboration is one of the guiding principles of LTC in the Catalog of Social Services [5]. In reality, it encounters multiple barriers (Poškutė *et al*., 2022).

### Design and sample

Data were collected through a survey of key LTC actors in Lithuania (home and residential care service providers, and governmental and municipal organizations responsible for LTC funding and regulating. We used total population sampling technique and aimed to invite all organizations that were reachable in our target population; therefore, we inquired and received information from the Ministry of Health, searched the websites of the Ministry of Social Security and Labor and municipalities, and this way, we gathered a comprehensive list of LTC providers in Lithuania. We also drew a list from the national business directory for cross-checking. After eliminating those whose contact details were not available, we ended up with the final list of 341 organizations and organizational units (e.g. ministries had several departments responsible for different aspects of LTC). Invitations to participate were sent to the heads of the 341 organizations/units, where contact details were available, or general contact emails, who were asked to share the survey link with their employees to reach different professional groups. If requested, we supplied respondents with paper questionnaires instead of online surveys. The invitation letters included information on the study aims, procedure and participation duration. Participation was voluntary and did not pose any potential risks to research participants, and fully anonymous in order to encourage the authenticity of the survey answers. 268 questionnaires were returned and after list-wise deletion due to missing values 265 were used in analysis, of which 117 (44.5%) were online questionnaires. Sample distribution per sector is provided in Table 1.

**Table 1:** Variables description, averages, and Cronbach α reliability scores

Variable name		Variable description	Mean (St.Dev.)*				Cronbach ɑ
			1. NGO (*N* = 44)	2. Private (*N* = 51)	3. Public (*N* = 170)	Total (*N* = 265)	
Dependent variables	*Knowledge and information transfer*	5-item scale (1–6)	4.43 (0.97)	4.45 (1.29)	4.57 (1.31)	4.52 (1.25)	0.896
Independent variables (*dimensions of social capital*)	*Structural* (number of relationships and interaction frequency)	No of groups with whom a person interacts * frequency of interaction (0–60)	12.61^2,3^ (10.44)	18.61^1,3^ (10.70)	23.63^1,2^ (9.47)	20.83 (10.68)	
	*Relational* (trust and reciprocity)	3-item scale (1–6)	3.97^2,3^ (0.89)	4.58^1^ (1.21)	4.5^1^ (1.13)	4.42 (1.13)	0.825
	*Cognitive* (LTC vision and values)	5-item scale (1–6)	5.55 (0.75)	5.71 (0.82)	5.81 (0.55)	5.75 (0.65)	0.875
Moderators	*Resource availability*	2-item scale (1–6)	2.38 (1.17)	2.99 (1.58)	2.86 (1.52)	2.8 (1.49)	0.806
	*Sector*	NGO, private, public (dummy variables)					
Control variable	*Type of activity*	1 – administrative, 0 – non-administrative					

**Note(s):** * One-Way Anova used to compare means. Superscript number next to a mean indicates significant difference at 0.01 level and a comparison group number: 1 NGO; 2 Private; 3 Public

**Source(s):** Authors’ work

### Measures

The questionnaire was developed to take into consideration LTC specifics. The items constituting the scales were revised for relevance, clarity, and comprehension, and adapted to LTC context in consultation with LTC experts (academics and practitioners).

*Social capital (independent variable).* There is no single measure of social capital, as different studies employ different definitions and dimensions; however, common measures include trust, norms and values, and network structure (Widén-Wulff and Ginman, 2004) and often rely on self-reporting and Likert scales (Miković *et al*., 2020). We measured social capital with 10 items reflecting LTC specifics:

1. **structural dimension** was operationalized as relationship number and frequency of interaction. The *number of relationships* was measured by asking respondents to indicate which of the 10 LTC actors (e.g. municipality staff, NGOs, etc.) they interacted with at work (min = 0, max = 10); and *frequency of interaction* – how often they interacted with each group (1 = never, 6 = more than once a week). Collinearity statistics of both variables remained within an acceptable range (tolerance = 33.2; VIF = 3.014). However, they were highly correlated (Pearson *r* = 0.817, *p* < 0.001) and the calculations of individual predictors in regression models were significantly affected by this correlation; therefore, a composite score of these two variables was used in the analysis (number of relationships multiplied by average frequency of interaction);
2. **relational dimension** was operationalized as *trust and reciprocity* and measured on a three-item scale (1 = totally disagree, 6 = totally agree). Sample item is *“If and when needed, other institutions would help us; therefore, we should help them as well”*;
3. **cognitive dimension** was operationalized as *LTC vision and values* and measured on a five-item scale, where respondents were asked to indicate to what extent specified LTC goals are important to their institution (1 = not important at all, 6 = highly important). Sample items are *“LTC service quality improvement”; “Life quality of older persons”.*

*Perceived knowledge and information transfer (hereinafter knowledge transfer),* the dependent variable, was measured by a five-item scale (1 = totally disagree, 6 = totally agree). Following prior research, existing scales were adapted to reflect the industry specifics. To do so, we identified major knowledge and information types shared and transfer activities through prior research review and consultations with experts in the field. Respondents were asked to indicate to what extent they shared information with other LTC actors and had information about them or from them. Sample item is *“We share our experience and knowledge with other institutions”.*

*Resource availability* (moderator) was measured on a two-item scale (1 = totally disagree, 6 = totally agree) adapted fromand He and Wei (2009), measuring availability of financial and time resources, major resources in inter-organizational knowledge and information transfer in LTC (Lim *et al.*, 2015). Sample item is *“We lack financial resources to collaborate with other institutions in LTC”* (for analysis, both items were reversed so that a higher score means higher resource availability).

*Sector* (moderator). In Lithuania, LTC providers are classified according to their founders – municipality (state), physical person (private for-profit and non-profit), and non-governmental (NGOs; mostly international and/or church-established organizations). We asked respondents to indicate if they worked for a public, private or non-governmental organization (NGO).

*Type of activity* (control variable) is a binary variable that was coded based on respondent job titles, distinguishing between an administrative (municipality staff; 18.1%) and a non-administrative (care providers; 81.9%) role. To limit its potential confounding effect, we included it as a covariate in hypothesis testing.

The scales were verified by using factor analysis (principal components analysis with varimax rotation), and also tested for internal consistency and Cronbach Alpha scores between 0.806 and 0.896 were regarded as having sufficient inter-relatedness for use in the analysis (Tavakol and Dennick, 2011). See Table 1 below for the description of variables, their means, standard deviations and internal consistency scores.

As we used single-source self-reported data and measured both dependent and independent variables at the same time, common method bias could be an issue (Podsakoff *et al*., 2003). A multi-wave study was not used because it would have affected the response rate, as participants were difficult to reach. Instead, several widely recommended procedures were used to minimize common method variance, i.e. item randomization, reverse scoring, and variation in wording, as well as guaranteed respondent anonymity. Results of Harman’s singe-factor test (Favero and Bullock, 2015) showed that one factor accounted for 23%, which is below the recommended threshold of 50%.

### Data analysis

To test the hypotheses, SPSS PROCESS macro 3.5 (Hayes, 2018) was used. Cohen (1992) guidance was followed to interpret effect strength (small if around 0.1, medium if around 0.3, and strong if around 0.5). The strength of correlation between independent variables (moderate positive effects where significant), condition index (less than 30) and VIF (less than 4) statistics raised no further concerns of multicollinearity issues. As different scales were used to measure the variables, mean-centered continuous variables were used in order to enhance result interpretability (Hayes, 2018).

## Results

Results of multiple regression analysis showed that the more respondents interacted with different actors (structural dimension, *b* = 0.26; *p* < 0.001) and the stronger was their trust and reciprocity in the relationships with them (relational dimension, *b* = 0.39; *p* < 0.001), the higher the level of perceived knowledge transfer they reported. However, the perceived importance of LTC vision and values was marginal and insignificant (cognitive dimension, *b* = 0.08, *p* > 0.05) when accounting for the other two dimensions of social capital (see Table 2). Therefore, hypothesis 1 was supported partially. Social capital explained approximately 27% of the results, with the relational dimension (medium effect) making the highest contribution to the total.

**Table 2:** Regression analysis results

	Hypothesis No.						
	1a,b,c	2a	2b	2c	3a	3b	3c
Structural	0.26*** (0.06)	0.19 (0.13)	0.32*** (0.06)	0.32*** (0.06)	0.23 (0.13)	0.32*** (0.06)	0.33*** (0.06)
Relational	0.39*** (0.06)	0.41*** (0.05)	0.04 (0.16)	0.4*** (0.05)	0.35*** (0.06)	0.01 (0.16)	0.37*** (0.05)
Cognitive	0.08 (0.06)	0.09 (0.06)	0.09 (0.06)	−0.15 (0.11)	0.10 (0.05)	0.10 (0.05)	−0.21 (0.13)
Private		−0.34 (0.21)	−0.26 (0.19)	−0.33 (0.18)	−0.27 (0.2)	−0.12 (0.19)	−0.33 (0.18)
Public		−0.33 (0.18)	−0.27 (0.17)	−0.35* (0.16)	−0.22 (0.18)	−0.18 (0.17)	−0.35* (0.16)
Resources					−0.15** (0.05)	−0.17*** (0.05)	−0.13* (0.06)
Structural * Private (w1)		−0.02 (0.18)			−0.06 (0.18)		
Structural * Public (w2)		0.22 (0.15)			0.14 (0.15)		
Relational * Private (w1)			0.41* (0.2)			0.35 (0.19)	
Relational * Public (w2)			0.43* (0.18)			0.36* (0.17)	
Cognitive * Private (w1)				0.33* (0.15)			0.51* (0.2)
Cognitive * Public (w2)				0.29* (0.14)			0.40* (0.16)
Structural * Resources					0.15** (0.05)		
Relational * Resources						0.10* (0.04)	
Cognitive * Resources							−0.12 (0.07)
Administrative position	−0.13 (0.15)	−0.15 (0.15)	−0.09 (0.15)	−0.09 (0.15)	−0.18 (0.15)	−0.12 (0.15)	−0.08 (0.15)
*R* ^2^	0.27	0.30	0.31	0.31	0.35	0.35	0.34
*N*	265	265	265	265	265	265	265

**Note(s):** Dependent variable: Knowledge and information transfer; Unstandardized coefficients (b) provided; standard errors in parenthesis; *p < 0.05. **p < 0.01. ***p < 0.001

**Source(s):** Authors’ work

**Figure 1:**
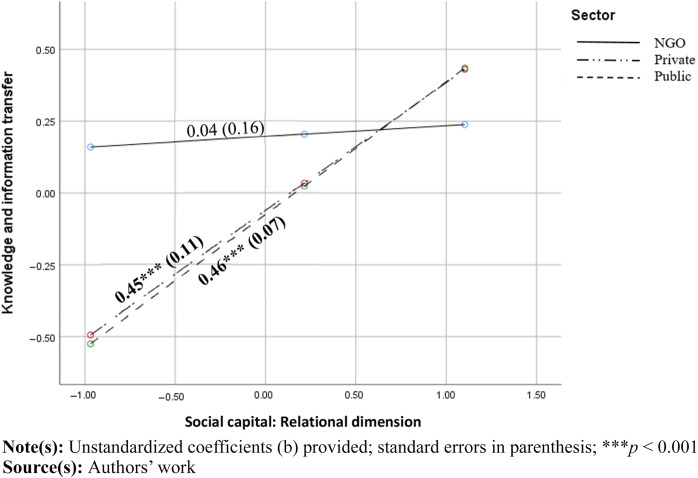
Moderating effect of sector on the relationship between relational dimension of social capital and knowledge and information transfer

Hypothesis 2 posited that the relationship between social capital and knowledge transfer among LTC actors is moderated by the sector. No significant sector effects were found in case of structural and cognitive dimensions. However, a medium moderating effect of the sector was identified in the relationship between relational dimension and perceived knowledge transfer (b_w1_ = 0.41 *p* < 0.05; b_w2_ = 0.43, *p* < 0.05). More specifically, higher levels of trust and reciprocity (relational dimension) predicted higher levels of perceived knowledge transfer in private and public sectors (*b* = 0.45 *p* < 0.001 and *b* = 0.46 *p* < 0.001 respectively); whereas in NGOs, the relationship was not significant (*b* = 0.04, *p* > 0.05, see Figure 1). This means that contrary to private and public sectors, there is no correlation between trust and reciprocity towards other organizations and the perceived extent to which NGOs share knowledge with others, which confirmed our hypothesis about a weaker relationship in NGOs. However, private and public sector slopes were rather similar, contrary to our expectation that the relationship will be the strongest in the private sector.

**Figure 2:**
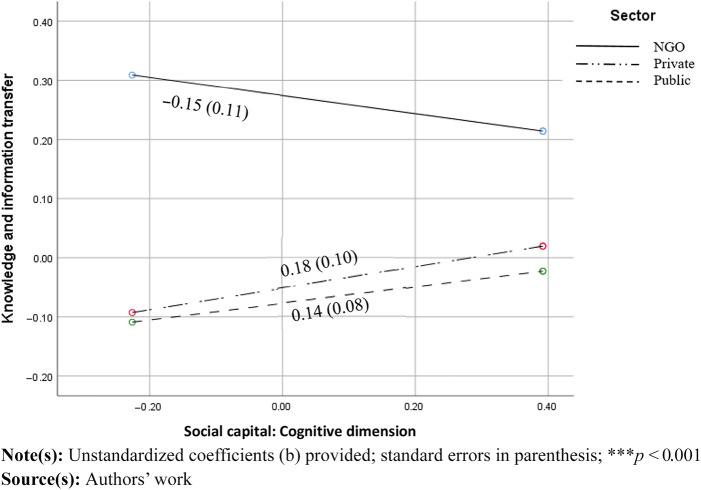
Moderating effect of sector on the relationship between cognitive dimension of social capital and knowledge and information transfer

Furthermore, the relationship between the cognitive dimension of social capital and perceived knowledge transfer was also moderated by sector (b_w1_ = 0.33 *p* < 0.05; b_w2_ = 0.29, *p* < 0.05). The relationship was significantly different in private and public sectors compared to NGOs; however, individual slopes were mutually non-significant (see Figure 2).

**Figure 3:**
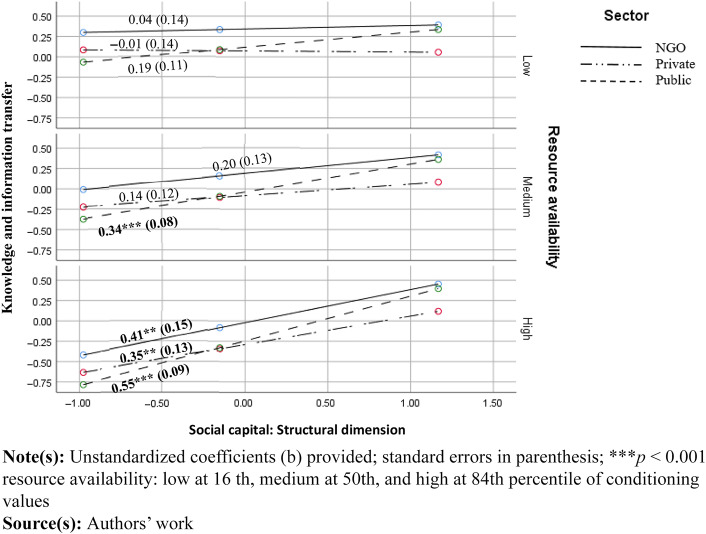
Conditional effects of social capital at the low, medium and low values of resources (structural dimension)

Hypothesis 3 proposed that the relationship between social capital and knowledge transfer depends on resource availability. Consistent with our hypothesis, the availability of resources moderated the relationship between structural and relational dimensions, and knowledge transfer (*b* = 0.15, *p* < 0.01 and *b* = 0.10, *p* < 0.05 respectively). More specifically, when resource availability was perceived as relatively low (16th percentile of conditioning values), the structural dimension of social capital had no effect on perceived knowledge transfer. However, when resource availability was perceived as high (84th percentile), the relationship was significant in all sectors, including NGOs (medium effects). Lastly, at a medium level of resource availability (50th percentile) the relationship was significant only in the public sector (see Figure 3 below).

**Figure 4:**
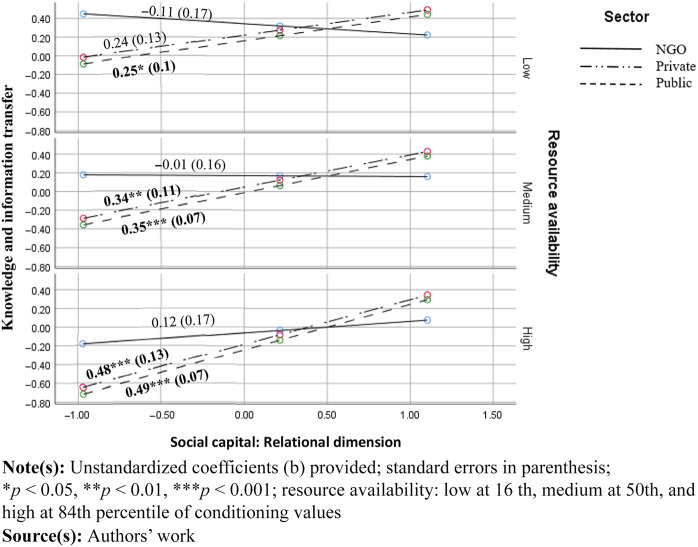
Conditional effects of social capital at the low, medium and low values of resources (relational dimension)

In case of the relational dimension of social capital, its relationship with knowledge transfer was not significant in NGOs at any level of resource availability. However, it was significant in private (medium to high effects at medium and high level of resource availability) and public sectors (increasing from small to high effect at all levels of resource availability, see Figure 4 below).

There was no significant interaction between resource availability and cognitive dimension (attitudes towards LTC values, *b* = −0.12, *p* > 0.05); therefore, hypothesis 3c was not supported.

To summarize, the above moderation analysis suggests that only within the structural dimension of social capital, higher resource availability increases perceived knowledge transfer in all three sectors. Whereas in private and public sectors (but not in NGOs), the higher the resource availability, the stronger the effect of structural and relational dimensions on perceived knowledge transfer.

**Figure 5:**
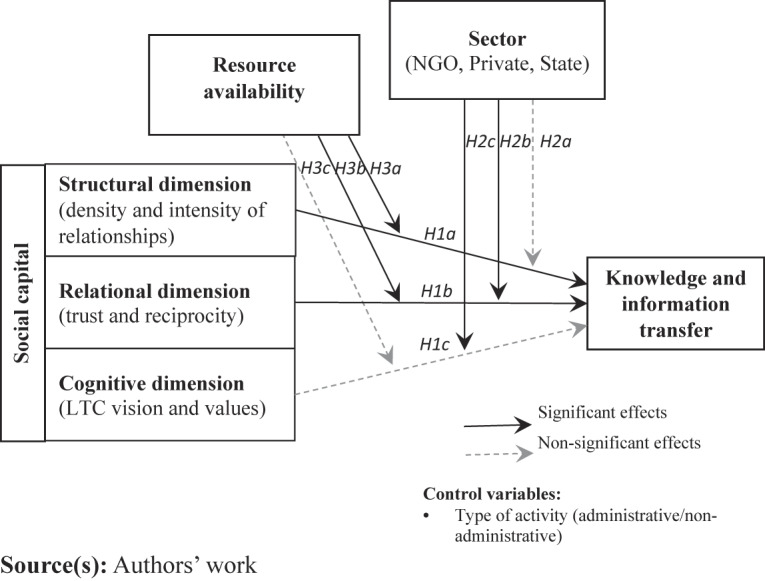
Final research model

Overall findings are summarized in Figure 5.

## Discussion and conclusions

This study investigated the relationship between social capital and perceived inter-organizational knowledge transfer in LTC and some boundary conditions.

### Theoretical implications

Results revealed a positive relationship between knowledge transfer and structural and relational dimensions of social capital, which corroborates prior research in other domains (e.g. Santos *et al*., 2023; Rodríguez-Aceves *et al*., 2023). However, contrary to expectations and prior research findings in other domains (e.g. Ganguly *et al*., 2019), the relationship with the cognitive dimension was insignificant. We speculate that the effect was insignificant because in LTC, a large network and ability to confide in its members are more relevant and indispensable for knowledge transfer than value congruence. Some individual level studies likewise failed to report significant relationship with cognitive capital (e.g. Akhavan and Mahdi Hosseini, 2016). This may also suggest that in LTC, other variables come into play that undermine this relationship, such as professional divergence in values and goals, etc. (Pearson and Watson, 2018; Auschra, 2018). It is therefore critical for future research to explore the potential effects of diverse professional groups working in LTC and their motivation to share knowledge with others, as in health and social care knowledge transfers are multilateral and involve multiple knowledge recipients (Radević *et al*., 2023). Another possibility for insignificant results in the case of cognitive dimension could be the interaction between social capital dimensions. For instance, Al-Tabbaa and Ankrah (2016) found that cognitive capital effects were indirect and mediated by the relational dimension, while Nadeem *et al*. (2021) found that trust moderated the relationship between shared goals and knowledge hiding. Thus, further research should explore these likely effects. In taking a three-dimensional perspective, this study contributes significantly to the larger knowledge management literature, as cognitive capital has been given little attention in studies of organizational social capital.

We also contribute to the knowledge management literature by exploring boundary conditions in the relationship between social capital and knowledge transfer, which, at least to our knowledge, have not received much attention. Our findings showed that NGOs benefit less from their social capital in knowledge transfer. More specifically, our results showed that higher trust and reciprocity lead to higher knowledge and information transfer in private and public sectors, but not in NGOs. We speculate that this is due to their lower levels of trust and reciprocity reported in comparison to public and private organizations in our study, which is in line with previous research on NGO perceptions of inferiority in comparison to other actors (Jang *et al*., 2016). In addition, NGOs may fear of losing autonomy and flexibility (Abendstern *et al*., 2018). In Lithuania NGOs are largely operated through younger volunteers who are often stigmatized as untrustworthy and the general public and politicians do not consider them as a serious actor (Poškutė *et al*., 2022). This may inhibit employee openness to knowledge transfer, while employee attitudes make a relevant enabler of this process in NGOs (Zbuchea *et al*., 2020).

We also found resource availability as another relevant boundary condition. Interestingly, in our study resource availability moderated the relationship between structural and relational dimensions and knowledge transfer in different ways. Importantly, it yielded some distinct findings for NGOs, where the effects of resource availability were less pronounced. This in turn suggests that in NGOs, knowledge and information transfer is motivated by other factors than in other sectors, and thus existing knowledge management research needs to be applied to NGOs with caution (Rathi and Given, 2017; Bloice and Burnett, 2016). For instance, employee altruism (Curado *et al*., 2023) and willingness to create social value for their wider communities and society (Bloice and Burnett, 2016) were found to be significant predictors of knowledge sharing in NGOs. Thus further comparative research is needed to identify distinct features of NGOs that might account for their engagement in knowledge transfer and the mechanisms driving it. NGOs may be substituting them with other types of resources, or there may be other contextual conditions (e.g. ties with external local communities; Schneider, 2009) facilitating knowledge exchange that could be explored in future studies.

*Implications for policy making*. Given LTC fragmentation, inadequacies in formal LTC supply and quality, and slow integrated care developments in many EU countries, cross-sectoral cooperation between major LTC actors plays a critical role. Knowledge transfer is a major facilitator of sustained cooperation. Our findings suggest that social capital facilitates knowledge transfer across the boundaries of different organizations, professions and sectors. To do so, organizations need to be provided with resources, network-building opportunities and measures facilitating frequent and continuous interactions to build trust and reciprocity.

Another important implication of our study concerns the required improvements regarding NGO integration in the LTC system, as their current engagement is insufficient. Our findings showed that in Lithuania NGOs have smaller networks and report lower levels of trust towards public and private organizations, which is also reflected in the disregard of their relevance by the politicians and general public (Poškutė *et al*., 2022). The insufficient cooperation between NGOs and government institutions and insufficient human and financial resources are acknowledged as a major constraint to their development in Lithuania [6]. Thus, immediate measures (including amendments in national legislation) need to be taken to resolve these issues, as NGOs can contribute significantly to higher LTC system efficiency and quality.

*Implications for practice*. To reap the benefits of inter-organizational cooperation, organizations first need to clearly communicate to their employees the aims and relevance of knowledge and information transfer both within and outside organizational boundaries. They should also provide their employees with networking possibilities and facilitate their more frequent interactions with their peers in other organizations; thus, contributing to the enhancement of both structural and relational dimensions of their social capital. In order to make it more efficient, employees also need to be provided with facilitating conditions, such as time and financial resources. In regard to NGOs, they should take a more proactive role in establishing and sustaining collaboration with other sectors and seek to improve their public image.

*Limitations and future research*. Our findings must be considered within the limitations of the study. Our study builds on self-reported measures. Future studies should use objective measures of knowledge and information transfer. We report findings from a single European country. As countries vary in LTC systems and involvement of private and non-governmental organizations, our results may not be generalizable across all European countries and call for research in other countries. Yet our findings provide relevant insights for countries with fragmented LTC systems, new EU Member states in particular that also share rather recent involvement of private organizations in LTC. In addition, we did not include informal caregivers in our study, as in Lithuania there is not a single organization representing them. In most of Europe, including Lithuania, a large part of LTC is carried out by informal carers (European Commission, 2021a); thus they make a relevant, but under-studied stakeholder (Hengelaar *et al*., 2018). Respectively, future studies should be conducted in countries were informal caregiver associations exist.

*Conclusions*. The purpose of this study was to disclose the relationship between social capital and inter-organizational knowledge and information transfer, as a critical facilitator of cooperation between LTC actors from different sectors. Our findings revealed a positive relationship between two social capital dimensions (network size number and strength, and trust and reciprocity) and knowledge and information transfer between LTC actors in Lithuania. In addition, we found that the relationship was partly moderated by the sector and resource availability.

## Notes

1. In LTC NGOs usually connote nonprofit organizations operating in the international context, largely relying on charitable contributions and the work of unpaid volunteers, while private non-profits typically refer to not-for-profit organizations founded by a private person relying on paid staff. In Lithuania private non-profits rely on service fees, part of which is covered by the municipality and the rest by the care recipient, as out-of-pocket spending, and their legal status is distinct from NGOs. The latter refer to organizations who have officially declared their activities as public interest seeking and who have been registered as non-governmental organizations in the National Register of Legal Entities.

2. Since 2009 Lithuanian population declined by 12% (Statistics Lithuania, 2022)

3. Aged 65+ comprise 21.6% (Statistics Lithuania, 2023)

4. 40.3% of older people are at risk of poverty or social exclusion (Eurostat, 2022)

5. Order No. A1-93 of the Minister of Social Security and Labor of the Republic of Lithuania on the approval of Catalog of Social Services.

6. Lithuanian Ministry of Social Affairs and Labor (Link to the website)

## References

[ref001] Abendstern, M., Hughes, J., Jasper, R., Sutcliffe, C. and Challis, D. (2018), “Care coordination for older people in the third sector: scoping the evidence”, Health and Social Care in the Community, Vol. 26 No. 3, pp. 314-329, doi: 10.1111/hsc.12420. 28118683

[ref002] Adler, P.S. and Kwon, S.-W. (2002), “Social capital: prospects for a new concept”, Academy of Management Review, Vol. 27 No. 1, pp. 17-40, doi: 10.2307/4134367.

[ref003] Akhavan, P. and Mahdi Hosseini, S. (2016), “Social capital, knowledge sharing, and innovation capability: an empirical study of R&D teams in Iran”, Technology Analysis and Strategic Management, Vol. 28 No. 1, pp. 96-113.

[ref004] Al-Tabbaa, O. and Ankrah, S. (2016), “Social capital to facilitate ‘engineered’ university–industry collaboration for technology transfer: a dynamic perspective”, Technological Forecasting and Social Change, Vol. 104, pp. 1-15, doi: 10.1016/j.techfore.2015.11.027.

[ref005] Alderwick, H., Hutchings, A., Briggs, A. and Mays, N. (2021), “The impacts of collaboration between local health care and non-health care organizations and factors shaping how they work: a systematic review of reviews”, BMC Public Health, Vol. 21, pp. 1-16, doi: 10.1186/s12889-021-10630-1. 33874927 PMC8054696

[ref006] Auschra, C. (2018), “Barriers to the integration of care in inter-organisational settings: a literature review”, International Journal of Integrated Care, Vol. 18 No. 1, p. 5, doi: 10.5334/ijic.3068. PMC5887071 29632455

[ref007] Bloice, L. and Burnett, S. (2016), “Barriers to knowledge sharing in third sector social care: a case study”, Journal of Knowledge Management, Vol. 20 No. 1, pp. 125-145, doi: 10.1108/jkm-12-2014-0495.

[ref008] Bourdieu, P. (1985), “The forms of capital”, in Richardson, J.G. (Ed.), Handbook of Theory and Research for the Sociology of Education, Greenwood, New York, pp. 241-258.

[ref009] Carlini, J., Lehman, K., Dharmesti, M. and Knox, K. (2023), “Maximizing value in healthcare partnerships: a case examining an inter-organizational relationship in the public and non-profit sectors”, Journal of Philanthropy and Marketing, Vol. 28 No. 3, e1796, doi: 10.1002/nvsm.1796.

[ref010] Cohen, J. (1992), “A power primer”, Psychological Bulletin, Vol. 112 No. 1, pp. 155-159, doi: 10.1037/0033-2909.112.1.155. 19565683

[ref011] Coleman, J.S. (1988), “Social capital in the creation of human capital”, American Journal of Sociology, Vol. 94, pp. 95-120, doi: 10.1086/228943.

[ref012] Curado, C., Henriques, P., Oliveira, M. and Martins, R. (2023), “Organisational culture as an antecedent of knowledge sharing in NGOs”, Knowledge Management Research and Practice, Vol. 21 No. 3, pp. 449-461, doi: 10.1080/14778238.2021.1908864.

[ref013] Derose, K.P. and Varda, D.M. (2009), “Social capital and health care access: a systematic review”, Medical Care Research and Review: MCRR, Vol. 66 No. 3, pp. 272-306, doi: 10.1177/1077558708330428. 19174538 PMC5661991

[ref014] Dickinson, H., Allen, K., Alcock, P., Macmillan, R. and Glasby, J. (2012), The Role of the Third Sector in Delivering Social Care, NIHR School for Social Care Research, London.

[ref015] Doornebosch, A.J., Smaling, H.J.A. and Achterberg, W.P. (2022), “Interprofessional collaboration in long-term care and rehabilitation: a systematic review”, Journal of the American Medical Directors Association, Vol. 23 No. 5, pp. 764-777.e2, doi: 10.1016/j.jamda.2021.12.028. 35065048

[ref016] Enjolras, B. and Sivesind, K.H. (2018), “The roles and impacts of the third sector in Europe”, in Enjolras, B., Salamon, L.M., Sivesind, K.H. and Zimmer, A. (Eds), The Third Sector as a Renewable Resource for Europe: Concepts, Impacts, Challenges and Opportunities, Springer International Publishing, Cham, pp. 95-124.

[ref017] European Commission (2021a), Long-term Care Report – Trends, Challenges and Opportunities in an Ageing Society, Vol. I, Publications Office, Luxembourg.

[ref018] European Commission (2021b), Long-Term Care Report. Trends, Challenges and Opportunities in an Ageing Society, Publications Office of the European Union, Luxembourg.

[ref079] Eurostat (2022), “Living conditions in Europe - poverty and social exclusion”, available at: Link to the website

[ref019] Favero, N. and Bullock, J.B. (2015), “How (not) to solve the problem: an evaluation of scholarly responses to common source bias”, Journal of Public Administration Research and Theory, Vol. 25 No. 1, pp. 285-308, doi: 10.1093/jopart/muu020.

[ref020] Ferrer-Serrano, M., Fuentelsaz, L. and Latorre-Martinez, M.P. (2022), “Examining knowledge transfer and networks: an overview of the last twenty years”, Journal of Knowledge Management, Vol. 26 No. 8, pp. 2007-2037, doi: 10.1108/jkm-04-2021-0265.

[ref021] Ganguly, A., Talukdar, A. and Chatterjee, D. (2019), “Evaluating the role of social capital, tacit knowledge sharing, knowledge quality and reciprocity in determining innovation capability of an organization”, Journal of Knowledge Management, Vol. 23 No. 6, pp. 1105-1135, doi: 10.1108/jkm-03-2018-0190.

[ref080] Gazley, B. (2010), “Why not partner with local government? Nonprofit managerial perceptions of collaborative disadvantage”, Nonprofit and Voluntary Sector Quarterly, Vol. 39 No. 1, pp. 51-76.

[ref022] Gonçalves, T., Curado, C. and Balle, A.R. (2022), “Psychosocial antecedents of knowledge sharing in healthcare research centers: a mixed-methods approach”, Journal of Health, Organisation and Management, Vol. 36 No. 1, pp. 1-23, doi: 10.1108/jhom-12-2020-0463. 34378370

[ref023] Gonçalves, T., Muñoz-Pascual, L. and Curado, C. (2024), “Is knowledge liberating? The role of knowledge behaviors and competition on the workplace happiness of healthcare professionals”, Journal of Health, Organisation and Management, Vol. 38 No. 4, pp. 469-493, doi: 10.1108/jhom-12-2022-0382. 38839779

[ref024] Hayes, A.F. (2018), Introduction to Mediation, Moderation, and Conditional Process Analysis: A Regression-Based Approach, Guilford Publications, New York.

[ref081] He, W. and Wei, K.-K. (2009), “What drives continued knowledge sharing? An investigation of knowledge-contribution and-seeking beliefs”, Decision Support Systems, Vol. 46 No. 4, pp. 826-838.

[ref025] Hemmert, M. (2019), “The relevance of inter-personal ties and inter-organizational tie strength for outcomes of research collaborations in South Korea”, Asia Pacific Journal of Management, Vol. 36 No. 2, pp. 373-393, doi: 10.1007/s10490-017-9556-6.

[ref026] Hengelaar, A.H., van Hartingsveldt, M., Wittenberg, Y., van Etten-Jamaludin, F., Kwekkeboom, R. and Satink, T. (2018), “Exploring the collaboration between formal and informal care from the professional perspective—a thematic synthesis”, Health and Social Care in the Community, Vol. 26 No. 4, pp. 474-485, doi: 10.1111/hsc.12503. 28990248

[ref027] Hujala, T. and Laihonen, H. (2021), “Effects of knowledge management on the management of health and social care: a systematic literature review”, Journal of Knowledge Management, Vol. 25 No. 11, pp. 203-221, doi: 10.1108/jkm-11-2020-0813.

[ref028] Hujala, T. and Laihonen, H. (2023), “Knowledge management in a regional integrated health and social care system”, Journal of Integrated Care, Vol. 31 No. 5, pp. 15-28, doi: 10.1108/jica-06-2022-0032.

[ref029] Jang, H.S., Feiock, R.C. and Saitgalina, M. (2016), “Institutional collective action issues in nonprofit self-organized collaboration”, Administration and Society, Vol. 48 No. 2, pp. 163-189, doi: 10.1177/0095399713513139.

[ref030] Jeon, S., Kim, Y.G. and Koh, J. (2011), “An integrative model for knowledge sharing in communities‐of‐practice”, Journal of Knowledge Management, Vol. 15 No. 2, pp. 251-269, doi: 10.1108/13673271111119682.

[ref031] Kim, M. and Peng, S. (2018), “The dilemma for small human service nonprofits: engaging in collaborations with limited human resource capacity”, Nonprofit Management and Leadership, Vol. 29 No. 1, pp. 83-103, doi: 10.1002/nml.21314.

[ref032] Koay, K.Y., Sandhu, M.S., Tjiptono, F. and Watabe, M. (2022), “Understanding employees' knowledge hiding behaviour: the moderating role of market culture”, Behaviour and Information Technology, Vol. 41 No. 4, pp. 694-711, doi: 10.1080/0144929x.2020.1831073.

[ref033] Kosklin, R., Lammintakanen, J. and Kivinen, T. (2023), “Knowledge management effects and performance in health care: a systematic literature review”, Knowledge Management Research and Practiceand, Vol. 21 No. 4, pp. 738-748, doi: 10.1080/14778238.2022.2032434.

[ref034] Laihonen, H., Kork, A.-A. and Sinervo, L.-M. (2024), “Advancing public sector knowledge management: towards an understanding of knowledge formation in public administration”, Knowledge Management Research and Practiceand, Vol. 22 No. 3, pp. 223-233, doi: 10.1080/14778238.2023.2187719.

[ref035] Lee, H., Ki-Hyun, U., Hughes, P., Hughes, M. and Shine, E.-K. (2023), “Understanding knowledge transfer in M&As: an integration of resource orchestration and social capital theories and evidence from UK acquiring firms”, European Management Journal, Vol. 41 No. 2, pp. 199-211.

[ref036] Levin, D.Z. and Walter, J. (2019), “Before they were ties: predicting the value of brand-new connections”, Journal of Management, Vol. 45 No. 7, pp. 2861-2890, doi: 10.1177/0149206318769994.

[ref082] Lim, S.Y., Jarvenpaa, S.L. and Lanham, H.J. (2015), “Barriers to interorganizational knowledge transfer in post-hospital care transitions: review and directions for information systems research”, Journal of Management Information Systems, Vol. 32 No. 3, pp. 48-74, doi: 10.1080/07421222.2015.1095013.

[ref037] Lørum, R.M. and Smith, F. (2024), “Strategies and practices for organizational learning in integrated care”, Journal of Health, Organization and Management, Vol. 38 No. 6, pp. 942-960, doi: 10.1108/jhom-11-2023-0342. 39198961

[ref038] Lyng, H.B., Ree, E., Strømme, T., Johannessen, T., Aase, I., Ullebust, B., Thomsen, L.H., Holen-Rabbersvik, E., Schibevaag, L., Bates, D.W. and Wiig, S. (2024), “Barriers and enablers for externally and internally driven implementation processes in healthcare: a qualitative cross-case study”, BMC Health Services Research, Vol. 24 No. 1, p. 528, doi: 10.1186/s12913-024-10985-2. 38664668 PMC11046894

[ref039] Maurer, I., Bartsch, V. and Ebers, M. (2011), “The value of intra-organizational social capital: how it fosters knowledge transfer, innovation performance, and growth”, Organization Studies, Vol. 32 No. 2, pp. 157-185, doi: 10.1177/0170840610394301.

[ref040] McFadyen, M.A. and Cannella Jr, A.A. (2004), “Social capital and knowledge creation: diminishing returns of the number and strength of exchange relationships”, Academy of Management Journal, Vol. 47 No. 5, pp. 735-746, doi: 10.2307/20159615.

[ref041] Miković, R., Petrović, D., Mihić, M., Obradović, V. and Todorović, M. (2020), “The integration of social capital and knowledge management–The key challenge for international development and cooperation projects of nonprofit organizations”, International Journal of Project Management, Vol. 38 No. 8, pp. 515-533, doi: 10.1016/j.ijproman.2020.07.006.

[ref042] Moshtari, M. and Vanpoucke, E. (2021), “Building successful NGO–business relationships: a social capital perspective”, Journal of Supply Chain Management, Vol. 57 No. 3, pp. 104-129, doi: 10.1111/jscm.12243.

[ref043] Nadeem, M.A., Liu, Z., Ghani, U., Younis, A. and Xu, Y. (2021), “Impact of shared goals on knowledge hiding behavior: the moderating role of trust”, Management Decision, Vol. 59 No. 6, pp. 1312-1332, doi: 10.1108/md-09-2019-1197.

[ref044] Nahapiet, J. and Ghoshal, S. (1998), “Social capital, intellectual capital, and the organizational advantage”, Academy of Management Review, Vol. 23 No. 2, pp. 242-266, doi: 10.2307/259373.

[ref045] Oh, Y. and Bush, C.B. (2016), “Exploring the role of dynamic social capital in collaborative governance”, Administration and Society, Vol. 48 No. 2, pp. 216-236, doi: 10.1177/0095399714544941.

[ref046] Pearson, C. and Watson, N. (2018), “Implementing health and social care integration in Scotland: renegotiating new partnerships in changing cultures of care”, Health and Social Care in the Community, Vol. 26 No. 3, pp. e396-e403, doi: 10.1111/hsc.12537. 29349854

[ref047] Pedersen, L.M., Jakobsen, A.L., Buttenschøn, H.N. and Haagerup, A. (2023), “Positive association between social capital and the quality of health care service: a cross-sectional study”, International Journal of Nursing Studies, Vol. 137, 104380, doi: 10.1016/j.ijnurstu.2022.104380. 36375309

[ref048] Podsakoff, P.M., MacKenzie, S.B., Lee, J.-Y. and Podsakoff, N.P. (2003), “Common method biases in behavioral research: a critical review of the literature and recommended remedies”, Journal of Applied Psychology, Vol. 88 No. 5, pp. 879-903, doi: 10.1037/0021-9010.88.5.879. 14516251

[ref049] Poškutė, V. and Greve, B. (2017), “Long‐term care in Denmark and Lithuania–A most dissimilar case”, Social Policy and Administration, Vol. 51 No. 4, pp. 659-675, doi: 10.1111/spol.12318.

[ref050] Poškutė, V., Kazlauskaitė, R. and Matonytė, I. (2022), “Stakeholder collaboration in long-term care of older people in Lithuania”, Health and Social Care in the Community, Vol. 30 No. 1, pp. 193-202, doi: 10.1111/hsc.13389. 33852735

[ref051] Putnam, R.D. (1993), “What makes democracy work?”, National Civic Review, Vol. 82 No. 2, pp. 101-107, doi: 10.1002/ncr.4100820204.

[ref052] Radević, I., Dimovski, V., Lojpur, A. and Colnar, S. (2023), “Quality of healthcare services in focus: the role of knowledge transfer, hierarchical organizational structure and trust”, Knowledge Management Research and Practice, Vol. 21 No. 3, pp. 525-536, doi: 10.1080/14778238.2021.1932623.

[ref053] Rathi, D. and Given, L.M. (2017), “Non-profit organizations' use of tools and technologies for knowledge management: a comparative study”, Journal of Knowledge Management, Vol. 21 No. 4, pp. 718-740, doi: 10.1108/jkm-06-2016-0229.

[ref054] Reynaers, A.M. and van der Wal, Z. (2018), “Do partners in PPPs view public and private management differently? Re‐examining Boyne’s hypotheses in the context of collaboration”, Australian Journal of Public Administration, Vol. 77 No. 2, pp. 294-308, doi: 10.1111/1467-8500.12254.

[ref055] Riedel, M., Kraus, M. and Mayer, S. (2016), “Organization and supply of long-term care services for the elderly: a bird's-eye view of old and new EU member states”, Social Policy and Administration, Vol. 50 No. 7, pp. 824-845, doi: 10.1111/spol.12170.

[ref056] Rodríguez-Aceves, L., Mojarro-Durán, B.I. and Rivera, A.E. (2023), “Enabling knowledge sharing through relational capital in a family business context”, Journal of the Knowledge Economy, Vol. 14 No. 3, pp. 2156-2186, doi: 10.1007/s13132-022-00955-6.

[ref057] Rydenfält, C., Persson, J., Larsson, R., Johansson, G. and Erlingsdóttir, G. (2024), “Inter-organizational home care nursing teams: a comparison of a region wide organizational change initiative with success factors identified by forerunners and team theory”, Home Health Care Management and Practice, Vol. 36 No. 3, pp. 151-156, doi: 10.1177/10848223231209926.

[ref058] Santos, R.F., Oliveira, M. and Curado, C. (2023), “The effects of the relational dimension of social capital on tacit and explicit knowledge sharing: a mixed-methods approach”, VINE Journal of Information and Knowledge Management Systems, Vol. 53 No. 1, pp. 43-63, doi: 10.1108/vjikms-05-2020-0094.

[ref059] Schneider, J.A. (2009), “Organizational social capital and nonprofits”, Nonprofit and Voluntary Sector Quarterly, Vol. 38 No. 4, pp. 643-662, doi: 10.1177/0899764009333956.

[ref060] Schulmann, K., Leichsenring, K. and Genta, M. (2014), “Social support and long-term care in Eu care regimes. framework conditions and initiatives of social innovation in an active ageing perspective”, *Overview Report. WP8 MoPAct Project*.

[ref061] Selsky, J.W. and Parker, B. (2005), “Cross-sector partnerships to address social issues: challenges to theory and practice”, Journal of Management, Vol. 31 No. 6, pp. 849-873, doi: 10.1177/0149206305279601.

[ref062] Simons, M., Goossensen, A. and Nies, H. (2022), “Interventions fostering interdisciplinary and inter-organizational collaboration in health and social care; an integrative literature review”, Journal of Interprofessional Education and Practice, Vol. 28, 100515, doi: 10.1016/j.xjep.2022.100515.

[ref063] Song, A.M., Saavedra Cisneros, A., Temby, O., Sandall, J., Cooksey, R.W. and Hickey, G.M. (2019), “On developing an inter-agency trust scale for assessing governance networks in the public sector”, International Public Management Journal, Vol. 22 No. 4, pp. 691-710, doi: 10.1080/10967494.2017.1370047.

[ref064] Spasova, S., Baeten, R., Coster, S., Ghailani, D. and Peña-Casas, R.V.B. (2018), Challenges in Long-Term Care in Europe, A Study of National Policies 2018, European Social Policy Network (ESPN), Brussels, European Commission.

[ref083] Statistics Lithuania (2022), “The population of Lithuania”, available at: Link to the website

[ref084] Statistics Lithuania (2023), “The Population of Lithuania”, available at: Link to the website

[ref065] Sunnemark, F., Lundqvist Westin, W., Al Saad, T. and Assmo, P. (2024), “Exploring barriers and facilitators to knowledge transfer and learning processes through a cross-departmental collaborative project in a municipal organization”, The Learning Organization, Vol. 31 No. 3, pp. 358-374, doi: 10.1108/tlo-01-2023-0003.

[ref066] Susha, I., Rukanova, B., Zuiderwijk, A., Gil-Garcia, J.R. and Gasco Hernandez, M. (2023), “Achieving voluntary data sharing in cross sector partnerships: three partnership models”, Information and Organization, Vol. 33 No. 1, 100448, doi: 10.1016/j.infoandorg.2023.100448.

[ref067] Tavakol, M. and Dennick, R. (2011), “Making sense of Cronbach’s alpha”, International Journal of Medical Education, Vol. 2, pp. 53-55, doi: 10.5116/ijme.4dfb.8dfd. 28029643 PMC4205511

[ref068] Taylor, S. and Todd, P.A. (1995), “Understanding information technology usage: a test of competing models”, Information Systems Research, Vol. 6 No. 2, pp. 144-176, doi: 10.1287/isre.6.2.144.

[ref069] Torchia, M., Calabrò, A. and Morner, M. (2015), “Public–private partnerships in the health care sector: a systematic review of the literature”, Public Management Review, Vol. 17 No. 2, pp. 236-261, doi: 10.1080/14719037.2013.792380.

[ref085] Triandis, H.C. (1977), Interpersonal Behavior, Brooks/Cole, Monterey.

[ref070] van der Schors, W., Roos, A.-F., Kemp, R. and Varkevisser, M. (2021), “Inter-organizational collaboration between healthcare providers”, Health Services Management Research, Vol. 34 No. 1, pp. 36-46, doi: 10.1177/0951484820971456. 33291978

[ref071] Van Wijk, R., Jansen, J.J. and Lyles, M.A. (2008), “Inter‐and intra‐organizational knowledge transfer: a meta‐analytic review and assessment of its antecedents and consequences”, Journal of Management Studies, Vol. 45 No. 4, pp. 830-853, doi: 10.1111/j.1467-6486.2008.00771.x.

[ref072] Widén-Wulff, G. and Ginman, M. (2004), “Explaining knowledge sharing in organizations through the dimensions of social capital”, Journal of Information Science, Vol. 30 No. 5, pp. 448-458, doi: 10.1177/0165551504046997.

[ref073] Woschke, T., Haase, H. and Kratzer, J. (2017), “Resource scarcity in SMEs: effects on incremental and radical innovations”, Management Research Review, Vol. 40 No. 2, pp. 195-217, doi: 10.1108/mrr-10-2015-0239.

[ref074] Yadav, S.K., Singh, S. and Prusty, S.K. (2025), “An integrative perspective on inter-organisational collaboration in healthcare: a modified total interpretive structural modelling approach”, Journal of Health, Organization and Management, Vol. ahead-of-print No. ahead-of-print, doi: 10.1108/jhom-05-2024-0203.

[ref075] Yang, T.-M. and Maxwell, T.A. (2011), “Information-sharing in public organizations: a literature review of interpersonal, intra-organizational and inter-organizational success factors”, Government Information Quarterly, Vol. 28 No. 2, pp. 164-175, doi: 10.1016/j.giq.2010.06.008.

[ref076] Yoshida, Y., Hirakawa, Y., Hong, Y.J., Mamun, M.R., Shimizu, H., Nakano, Y. and Yatsuya, H. (2024), “Factors influencing interprofessional collaboration in long-term care from a multidisciplinary perspective: a case study approach”, Home Health Care Services Quarterly, Vol. 43 No. 4, pp. 239-258, doi: 10.1080/01621424.2024.2331452. 38521999

[ref077] Zbuchea, A., Ivan, L., Petropoulos, S. and Pinzaru, F. (2020), “Knowledge sharing in NGOs: the importance of the human dimension”, Kybernetes, Vol. 49 No. 1, pp. 182-199, doi: 10.1108/k-04-2019-0260.

[ref078] Zhao, L. and Detlor, B. (2023), “Towards a contingency model of knowledge sharing: interaction between social capital and social exchange theories”, Knowledge Management Research and Practice, Vol. 21 No. 1, pp. 197-209, doi: 10.1080/14778238.2020.1866444.

